# Malaria intervention scale-up in Africa: effectiveness predictions for health programme planning tools, based on dynamic transmission modelling

**DOI:** 10.1186/s12936-016-1461-9

**Published:** 2016-08-18

**Authors:** Eline Korenromp, Guy Mahiané, Matthew Hamilton, Carel Pretorius, Richard Cibulskis, Jeremy Lauer, Thomas A. Smith, Olivier J. T. Briët

**Affiliations:** 1 Avenir Health, Geneva, Switzerland; 2 Avenir Health, Glastonbury, USA; 3 World Health Organization Global Malaria Programme, Geneva, Switzerland; 4 World Health Organization Health Systems Governance and Financing dept., Geneva, Switzerland; 5 Swiss Tropical and Public Health Institute, Basel, Switzerland; 6 University of Basel, Basel, Switzerland

**Keywords:** Malaria, Prevention, Treatment, Vector control, Mortality, Morbidity, Health impact, Insecticide-treated mosquito nets, Indoor residual spraying, Programme planning, Modelling

## Abstract

**Background:**

Scale-up of malaria prevention and treatment needs to continue to further important gains made in the past decade, but national strategies and budget allocations are not always evidence-based. Statistical models were developed summarizing dynamically simulated relations between increases in coverage and intervention impact, to inform a malaria module in the Spectrum health programme planning tool.

**Methods:**

The dynamic *Plasmodiumfalciparum* transmission model OpenMalaria was used to simulate health effects of scale-up of insecticide-treated net (ITN) usage, indoor residual spraying (IRS), management of uncomplicated malaria cases (CM) and seasonal malaria chemoprophylaxis (SMC) over a 10-year horizon, over a range of settings with stable endemic malaria. Generalized linear regression models (GLMs) were used to summarize determinants of impact across a range of sub-Sahara African settings.

**Results:**

Selected (best) GLMs explained 94–97 % of variation in simulated post-intervention parasite infection prevalence, 86–97 % of variation in case incidence (three age groups, three 3-year horizons), and 74–95 % of variation in malaria mortality. For any given effective population coverage, CM and ITNs were predicted to avert most prevalent infections, cases and deaths, with lower impacts for IRS, and impacts of SMC limited to young children reached. Proportional impacts were larger at lower endemicity, and (except for SMC) largest in low-endemic settings with little seasonality. Incremental health impacts for a given coverage increase started to diminish noticeably at above ~40 % coverage, while in high-endemic settings, CM and ITNs acted in synergy by lowering endemicity. Vector control and CM, by reducing endemicity and acquired immunity, entail a partial rebound in malaria mortality among people above 5 years of age from around 5–7 years following scale-up. SMC does not reduce endemicity, but slightly shifts malaria to older ages by reducing immunity in child cohorts reached.

**Conclusion:**

Health improvements following malaria intervention scale-up vary with endemicity, seasonality, age and time. Statistical models can emulate epidemiological dynamics and inform strategic planning and target setting for malaria control.

**Electronic supplementary material:**

The online version of this article (doi:10.1186/s12936-016-1461-9) contains supplementary material, which is available to authorized users.

## Background

Effective malaria prevention and treatment interventions have been scaled-up substantially with increasing national and donor funding since the early 2000s. Between 2000 and 2015, malaria incidence rates fell 37 % globally, and malaria mortality rates by 60 %, with even greater declines in Africa, the highest-burden region [1]. This was likely a combined result of improved malaria control and other factors independent of interventions [2].

To sustain these improvements, the World Health Organization (WHO) Global Technical Strategy for Malaria recommends further scale-up to universal coverage with suitable preventive and curative interventions [3]. Funding for malaria has now plateaued, however, placing more emphasis on prioritizing interventions with the most impact. While most countries focus on WHO-recommended proven effective interventions, national strategies and plans vary considerably in budget allocations across interventions, and rationales for mixes of interventions are often not explicit [4]. National malaria control strategies and budget allocations should be evidence-based and explicitly justified. Field trials can directly inform only a sub-set of decisions, while separately parameterizing and analysing dynamical transmission models tailored to each individual setting and policy option is prohibitively complex.

For HIV/AIDS, tuberculosis, family planning and other health areas, strategic decision-making is supported by simple programme planning tools that project the impact and cost of user-defined scale-up scenarios. One such tool is the Spectrum suite of policy models, used by over 120 low and middle-income countries for estimation of burdens, trends, service needs and programme impact for family planning, HIV/AIDS and tuberculosis [5–8]. As of 2015, Spectrum did not have a malaria module, though a simple linear coverage-impact function included in the Lives Saved Tool (LiST) can model impacts of a sub-set of malaria interventions on under-5 mortality [9–11].

This article reports improved coverage-impact relationships developed for a Spectrum malaria impact module. Impacts on both morbidity and mortality of scale-up of insecticide-treated mosquito nets (ITNs), indoor residual spraying (IRS), effective management of uncomplicated malaria cases (CM) and seasonal malaria chemoprophylaxis (SMC) were analysed using OpenMalaria, an individual-based stochastic model of *Plasmodium falciparum* infection and disease dynamics in human populations exposed to mosquitoes, which has been fitted to extensive data on age and exposure patterns of prevalence and disease in sub-Sahara African settings with stable endemic malaria [12, 13]. The simulated impacts (in different age groups) were summarized using regression models, and the results compared with previous international consensus estimates and key empirical data. The validity, precision and accuracy of the resulting statistical relationships were considered with a view to their use for national programme planning.

## Methods

### Definition of interventions

For each of the four interventions coverage-impact relationships were simulated for scale up with coverage varying between 0 and 80 %.

Effective coverage of CM, defined as ‘adherence to and completion of a full course of a recommended treatment with a good-quality anti-malarial medication’, was expressed in terms of coverage within 14 days of onset of the episode, and modelled as described in [14]. A value of 48 % was assumed for effective coverage of appropriate care for severe cases in all simulations [15].

IRS was simulated with the long-acting insecticide Actellic CS, which kills mosquitoes up to 12 months or longer [16], at the beginning of each year i.e. before the peak transmission season. Unlike for other interventions, for IRS the coverage simulated was either 0 or 80 % of the population at risk protected, without any intermediate values, to reflect policy-making and practice of IRS implementation at district level.

ITNs were modelled as pyrethroid-impregnated long-lasting nets, assuming full mosquito susceptibility (type Zeneti) [17, 18]. The proportion of mosquitoes whose biting is potentially impeded by ITNs and IRS (πi), based on patterns of night-time versus day-time biting and human behaviours, was set at 0.65 [19, 20]. ITN coverage was defined as the proportion of people of any age at risk of malaria who slept under an ITN the previous night. In OpenMalaria, this coverage was achieved simulating annual deployments of ITNs, each assumed to be effective for 1 year without decay.

SMC was represented as three rounds of presumptive anti-malarial treatment per year, at start of months 2, 3 and 4, which correspond to the peak transmission season, to a specified proportion of resident children 3–59 months old, in line with the WHO recommendation of a maximum of four courses delivered at monthly intervals in areas with highly seasonal malaria transmission across the Sahel region [21, 22]. The simulated drug was amodiaquine plus sulfadoxine-pyrimethamine, which clears blood stage infections, and has a pre-erythrocytic prophylactic effect. While in most countries, four monthly rounds are scheduled, three monthly courses were modeled to account for children missing a course. The simulation selected these children randomly from the simulated population as a cohort, with the specified coverage proportion of children receiving all three SMC rounds, and the remaining non-covered children receiving no SMC at all. In OpenMalaria, SMC acts as an effective short-acting drug treatment, clearing any malaria infections (whether symptomatic or not) over the 10 days following drug administration, and lowering acquired immunity, similar to the effect of vaccination [23].

### Simulation of status quo

Simulated populations had an age distribution as in rural Tanzania [24]. All simulations included a low level of imported infections (1 per 1000 people per year) to prevent stochastic malaria extinction. Six different variants (calibrations) of OpenMalaria were used [25], differing in assumptions about immunity decay, heterogeneity in transmission, and co-morbidity [13]. These contributed uncertainty to the statistical impact functions. All possible combinations of parameter values listed in Table 1 comprise a total of 165,888 scenarios. Each scenario was simulated once for a population of 50,000 individuals, which was judged adequate to minimize undesired stochastic noise in case incidence. Simulations were run using OpenMalaria Schema version 32 [26].

**Table 1:** Design of simulations in OpenMalaria dynamic transmission model

Parameter	Parameter values specifying simulations
Transmission seasonality Coefficient of variation in EIR over a year	Low seasonal: 0.121 Moderately seasonal: 1.31 Highly seasonal: 2.66
Pre-intervention annual EIR (infectious bites per person per year) during simulation’s warm-up phase before IRS intervention starts	1, 3, 10, 30, 100 and 300
ITN coverage: people sleeping under ITN the previous night	Initial: 0, 30, 60 % Target: 0, 30, 60, 80 %
IRS coverage: people protected	Initial: 0, 80 % Target: 0, 80 %
Case management: uncomplicated cases treated effectively	Initial: 0, 30, 60 % Target: 0, 30, 60, 80 %
Seasonal malaria chemoprophylaxis: children 3–59 months old receiving three courses within a malaria season	Initial: 0 % Target: 0, 30, 60, 80 %

Differing endemicity was simulated by varying both the level and seasonality of the annual entomological inoculation rate (EIR). Six baseline transmission levels, covering the EIR range from 1 to 300 infectious bites per person-year (Table 1), were modelled. Seasonality in transmission was parameterized as the coefficient of variation (CV) in EIR over a year, defined as the standard deviation divided by the year-average of monthly EIR [27], and simulated at three values (Table 1):

- Low seasonality, as the 5th percentile of CV across malaria-endemic sub-Saharan Africa, based on a map of seasonality estimates provided by the Malaria Atlas Project (MAP) [2, 28]; such a seasonality might be expected in Equatorial Guinea;
- High seasonality, as the 95th percentile of CV, representing for example northern Burkina Faso;
- Intermediate seasonality, corresponding to the 50th percentile.

Populations were simulated with long warm-up phases so that intervention scale-up started at endemic equilibrium prevalence of infection with *P. falciparum* (*Pf*PR) with the assigned EIR and initial coverage of CM (Table 1) applied throughout the warm-up [29]. The simulations were run forward with the 2015 coverage of IRS added in, to reach a new approximate steady state. ITN scale-up to the initial (2015) coverage was simulated as a linear increase from 0 % of people sleeping under an ITN in 2004 up to the defined initial coverage in 2014 (Table 1) in line with ITN scale-up patterns in sub-Saharan Africa [1].

### Simulation of scale-up

Target coverage levels of interventions are given in Table 1. The 80 % maximum coverage target corresponds to projections used by WHO’s Malaria Global Technical Strategy [3], with universal coverage of core malaria interventions interpreted as 80–90 % for ITNs, 80–90 % for SMC as well as 80–90 % for artemisinin-based treatment of laboratory-confirmed malaria cases in the public sector [30]. Scale-up to target coverages was implemented as a one-off step increase at the start of 2016, maintained until the end of 2025.

### Health outcomes

The outcomes analysed were:

- Incidence of malaria episodes (including uncomplicated and severe episodes), in 0–4 year olds, 5–14 year olds and 15+ year olds;
- Malaria-attributable mortality rate, in 0–4 year olds, 5–14 year olds and 15+ year olds;
- *Pf*PR in 2–9 year olds, which is the age group for which most *Pf*PR data are available.

Impacts were analysed as proportional reductions in case incidence, malaria-attributable mortality and *Pf*PR, relative to ‘counterfactual’ scenarios with zero coverage of the intervention concerned over the same time period, to characterize the full impact of each intervention. Impacts were modelled separately for three time horizons: 1–3 years following intervention scale-up, and 4–6 years and 8–10 years after intervention scale-up in year ‘1’ (denoted 2016 in calendar time). Simulation outcomes were recorded at monthly intervals, and aggregated to annual averages or totals for statistical modelling.

### Statistical analysis

Relationships between intervention coverage, endemicity and the following health burden outcomes in OpenMalaria simulations were fitted using polynomial regressions. Outcome variables (i.e. dependent variables) were:

- Incidence rate of malaria episodes/cases (uncomplicated and severe), in 0–4 year olds, 5–14 year olds and 15+ year olds in the population (whether or not diagnosed in a health facility);
- Direct malaria-attributable death rate, in 0–4 year olds, 5–14 year olds and 15+ year olds;
- *Pf*PR (a ratio), in 2–9 year olds.

Each outcome in each age group was assessed separately for three different time periods within the time horizon most relevant to strategic planning, i.e. 10 years from intervention start. The three time periods were taken as multiple-year averages, to reduce stochastic noise: years 1–3, years 4–6 and years 8–10.

Outcome variables were logit-transformed. Outcomes that can take values greater than one were rescaled by dividing each value by the maximum value across all simulations. To allow well-defined regressions on logit-transformed outcomes, all zero outcomes (i.e. an average of zero over years 1–3, years 4–6 or years 8–10) were replaced by half of the minimum rate in any other simulated scenario-year for that outcome. This was done for <0.01 % of simulation-year data points for *Pf*PR and case incidence in all time periods and age groups, for 18–24 % of for malaria-attributable mortality data points in 0–4 year olds (across the three horizons), for 7–11 % of data points for 5–14 year olds and for 5–10 % of data points for 15+ year olds.

Explanatory (i.e. independent) variables (all continuous; Additional files 1, 2) were:

- Simulated *Pf*PR in 2–9 year olds averaged over 2000–2002, and *Pf*PR in 2–9 year olds averaged over 2000–2002 to the power of one-third (1/3);
- Simulated annual EIR, averaged over 2000–2002 at a log10 scale, as well as a coefficient for the one-third power of simulated EIR at a log10 scale;
- OpenMalaria model variant;
- Seasonality CV;
- Initial (2015) coverages for ITN, IRS and CM;
- Target coverages (2016 and onwards) for ITN, IRS, CM and SMC.

Model and predictor variable selection was done using Akaike’s Information Criterion (AIC) [31], for the impact functions for each of the seven outcomes at the 2019–2021 time horizon. All potential predictors, including simulated *Pf*PR and EIR, and their second-order (quadratic) terms, together with the interaction effects were included in an initial model. The stepwise (bidirectional elimination) AIC procedure was then applied to select models. The seven resulting regression structures for this (years 4–6) time horizon were then imposed and applied to the corresponding seven outcomes for years 1–3, and to the seven outcomes for years 8–10 models for each of the health outcome and age group combinations, so as to obtain standardized statistical impact models for each of the seven combinations of health outcomes and age groups, that are easily interpreted and compared in terms of the pattern over the overall 10-year horizon.

Statistical modelling was performed using the R-statistical package [32] version 3.1.3, using the linear model function ‘lm’ in R. The R code is available from Avenir Health upon request.

Both the OpenMalaria model variant and 2000–2002 annual EIR were retained as explanatory variables in the selected statistical models. However, for making predictions, 2000–2002 annual EIR and the most realistic OpenMalaria model variant are not known at country and province levels. A further statistical model was used to predict 2000–2002 annual EIR based on the country or province-level predictor variables (Additional file 2, sheet ‘EIR coefficient + p value’). Health impact predictions, using EIR thus estimated (with R^2^ of 98 %), were made for each of the six OpenMalaria model variants, and results for each intervention scenario presented and analysed as the average of these six predictions.

### Statistical predictions

The selected statistical models and their parameterizations were applied to predict health impacts of intervention scale-up (and scale-down) for four hypothetical provinces, covering the range of endemicity situations occurring in sub-Saharan Africa with respect to baseline endemicity and seasonality. Seasonality values at the 2.5th and 97.5th percentiles of seasonality CVs across Admin1 units in sub-Saharan Africa [2, 28] were selected; for each of these two seasonality CV values, selected *Pf*PR values were the 10th and 90th percentile of distributions of simulated *Pf*PR in 2–9 years averaged over 2000–2002 in the subset of OpenMalaria scenarios with 0 % baseline coverage of all interventions: i.e. for a low seasonality CV of 0.2, *Pf*PR values of 11 and 82 %; and for the high seasonality CV of 2.5, *Pf*PR values of 0.3 and 71 %, respectively.

Prediction results were presented as proportional reductions in the burden rate, based on burden rates predicted following intervention scale-up, compared with burden rates at the same time horizon for a ‘counterfactual scenario’ without that intervention’s (or combination of interventions) scale-up. Unless otherwise indicated, predictions assumed 0 % coverage of all interventions before 2016. Proportional reductions were the focus outcome instead of absolute reductions, because OpenMalaria burden levels were not calibrated on burden levels as estimated by the WHO, which have different definitions. The Spectrum programme planning tool will apply the predicted proportional reductions to WHO ‘baseline burden’ estimates to predict future burdens as a function of intervention scale-up.

### Assessing internal and external validity of statistical models

Internal validity of the statistical models was assessed based on adjusted coefficient of determination (R^2^), for the selected models described above and several alternative models, differing in the treatment of simulated zero outcomes, in the transformation applied to simulation outcomes before regressions, or in predictor variables included.

For each model, furthermore, out-of-sample predictions were performed, by drawing random samples of 100,000 simulations to inform the regression functions. Prediction errors were then assessed by estimating the mean squared error (MSE), evaluated using the remaining 65,888 simulations. This process was repeated 25 times and the average of their MSE was expressed and evaluated relative to the variance in the simulated health outcome of interest.

External validity was assessed by comparing patterns of predicted proportional health impacts as a function of endemicity, age, time, coverage levels, and interactions among interventions with earlier simulations by OpenMalaria and other dynamic transmission models.

In addition, predictions were performed for three cluster-randomized ITN trials conducted in the 1990s in Western Kenya, coastal Kenya and Ghana that observed estimated community-level ITN impacts on parasite infection prevalence, case incidence and mortality in children under-5 years [33], which are considered the gold standard for earlier global, regional and country-level ITN impact estimations [10, 11, 34–36]. These predictions, in contrast to those for the hypothetical provinces, used separate regression functions for only years 1 and 2 following ITN scale-up, to match the two-year duration of the trials. Predictor variables for the three trial predictions were based on endemicity as estimated by MAP, and trial data on coverage of ITNs and CM (Additional file 3). For mortality, since trials used as key observed outcome all-cause under-5 mortality, trial predictions included not only direct malaria-attributed mortality, but also indirect malaria-related mortality, which in OpenMalaria simulations for African settings occurs at similar rates as direct malaria-attributable mortality [15]. For comparison with trial data, predicted direct and indirect malaria mortality reductions were converted into all-cause under-5 mortality reductions and an overall malaria-related mortality reduction applying the one-cause-one-death framework and calculation proposed by the UN Child Epidemiology Reference Group [10, 11] (Additional file 3).

## Results

### Statistical models: internal validity

Statistical impact functions fitted OpenMalaria simulations reasonably well, with the proportion of explained variation (adjusted R^2^, in the logit scale) ranging 94–97 % for *Pf*PR and 86–97 % for case incidence across the three time horizons and age groups (Additional file 2). R^2^s were 74–95 % for malaria mortality, a slightly less good fit, likely reflecting stochastic noise in the simulations associated with small numbers of deaths. For each outcome, R^2^s were similar across the three horizons, but generally higher for younger age groups (where larger numbers of burden events reduced stochastic noise).

### Statistical models: epidemiological patterns

**Fig. 1:**
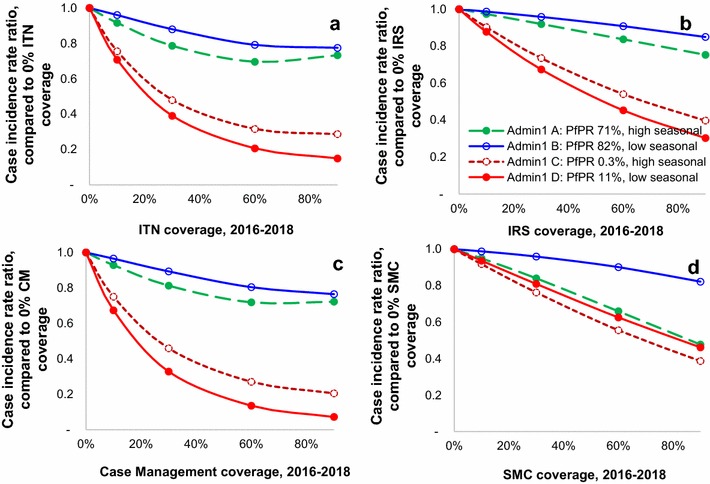
Proportional reductions in malaria case incidence in 0–4-year-olds, 1–3 years after intervention scale-up. **a** ITNs, **b** IRS, **c** case management, **d** SMC. The four hypothetical provinces had seasonality values at the 2.5th and 97.5th percentiles of seasonality CVs across Admin1 units in sub-Saharan Africa [2, 28]; for each of these two seasonality CV values, selected *Pf*PR values were the 10th and 90th percentile of distributions of simulated *Pf*PR in 2–9 years averaged over 2000–2002 in the subset of OpenMalaria scenarios with 0 % initial coverage of all interventions: i.e. for a low seasonality CV of 0.2, *Pf*PR values of 11 and 82 %; and for the high seasonality CV of 2.5, *Pf*PR values of 0.3 and 71 %, respectively

**Fig. 2:**
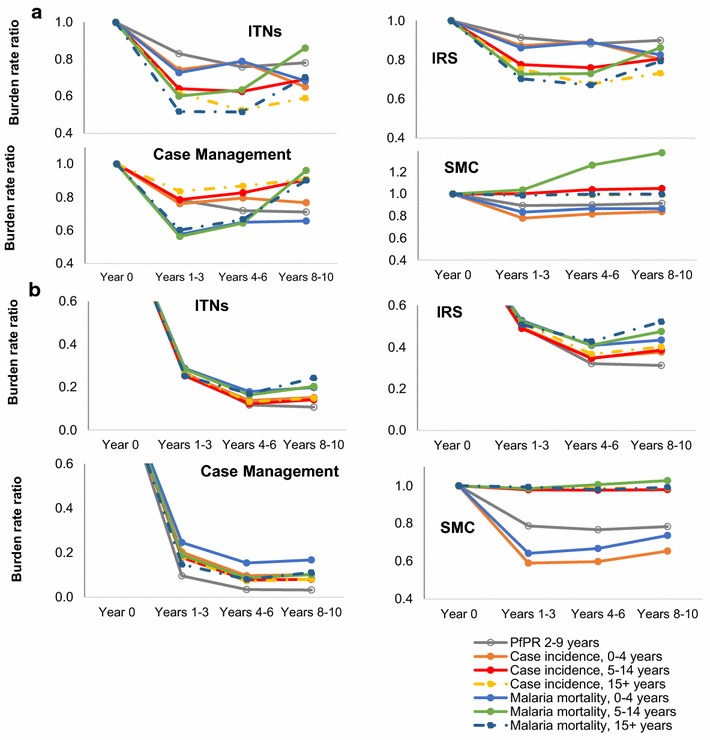
Impact of up scaling coverage from 0 to 60 % for Admin1 units with **a** high; **b** low *Pf*PR. Estimates from statistical models, as averages for **a** two Admin1 units with high (71 and 82 %) baseline *Pf*PR in 2–9 years; **b** two Admin1 units with low (0.3 and 11 %) baseline in *Pf*PR in 2–9 years

Statistical regression functions predicted large impacts following scale-up of especially CM and ITNs, followed by IRS, across all three age groups and across the three time horizons (Figs. 1, 2). For any given effective population coverage, CM averted most infections, episodes and deaths, with somewhat lower (similar) impacts for ITNs and IRS. Impacts of SMC were largely limited to the targeted children. The ranking in proportional burden reductions across the four interventions was stable across hypothetical Admin1 units that differ in baseline endemicity and seasonality (Fig. 1).

Proportional burden reductions were larger at lower baseline *Pf*PR (red lines in Figs. 1, 2) and, within low-endemic settings, larger at lower seasonality (solid red lines in Fig. 1). An exception is SMC, for which (as expected) proportional impacts were always larger under high seasonality, at both low and high baseline *Pf*PR.

Proportional burden reductions were typically slightly larger for parasite prevalence and case incidence than for malaria mortality (Fig. 2).

Across interventions, the maximum impact (lowest burden rate ratios) were generally achieved 4–6 years after reaching target coverage levels, with some partial rebounds over years 7–9, notably for mortality in children 5–14 years of age, due to reduced acquired immunity. For the simulated one-off coverage increases implemented instantaneously and then sustained, 70–90 % of long-term impacts were reached within the 1–3 year horizon (Fig. 2).

Across age groups, the proportional burden reductions achieved by ITNs, IRS and CM were similar at 1–6 years after scale-up, although at 7–9 years after scale-up the mortality reductions were slightly less in older age groups.

SMC, in contrast, reduced burdens of *Pf*PR, case incidence and mortality only in the targeted age group of 0–4 year olds, and slightly increased mortality for 5–14 year olds starting from years 4–6 after scale-up onward, reflecting reduced acquired immunity among the cohort of children who received SMC at age 0–4 years. Impacts of SMC were highest in areas with high malaria seasonality (dashed lines in Fig. 1d), but also considerable in areas with non-seasonal malaria (solid lines in Fig. 1d).

For CM and especially ITNs (the interventions with the largest impact), the proportional burden reduction for a given coverage percentage increase started to gradually diminish with increasing coverage, noticeably from a coverage of about 40 % (Fig. 1). In contrast, SMC impacts, which were smaller, increased linearly throughout the coverage increase. IRS impacts were near-linear with coverage as well, which may, however, be an artifact of the regression being based on only two simulated extreme coverage levels.

**Fig. 3:**
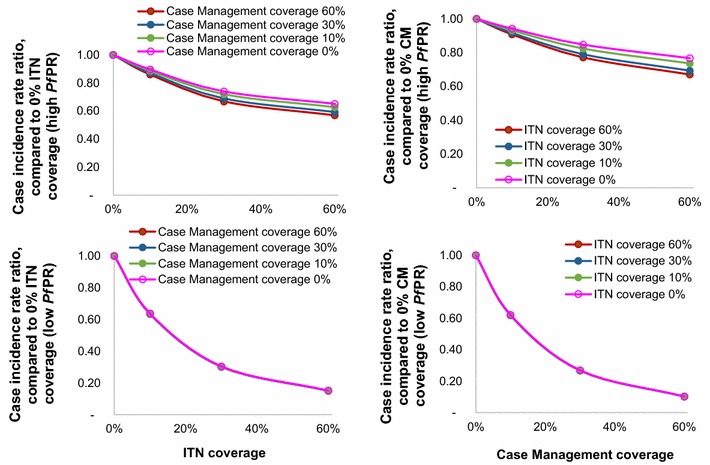
Proportional reductions in case incidence in 0–4-year-olds 8–10 years following ITN and/or CM scale-up. Estimated by statistical models, averaged over four hypothetical Admin1 units. *Left* ITN scale-up from 0 to 60 %, by level of target CM coverage; *Right* CM scale-up 0–60 %, by level of target ITN coverage; *Top* Average over two Admin1 units with high (71 and 82 %) baseline *Pf*PR in 2–9 years; *Bottom* Average over two Admin1 units with low (0.3 and 11 %) baseline *Pf*PR in 2–9 years

When combining CM and ITNs, their overall impact was slightly larger than the sum of the individual interventions for settings with high baseline endemicity (Fig. 3), reflecting that proportional burden reductions increase with decreasing endemicity (Fig. 1), and combined interventions more powerfully reduce endemicity from high baseline endemicity. For settings with low baseline endemicity, in contrast, the proportional impact of either intervention did not vary with the coverage level of the other intervention.

### External validity: predicted impacts against ITN trial data

**Fig. 4:**
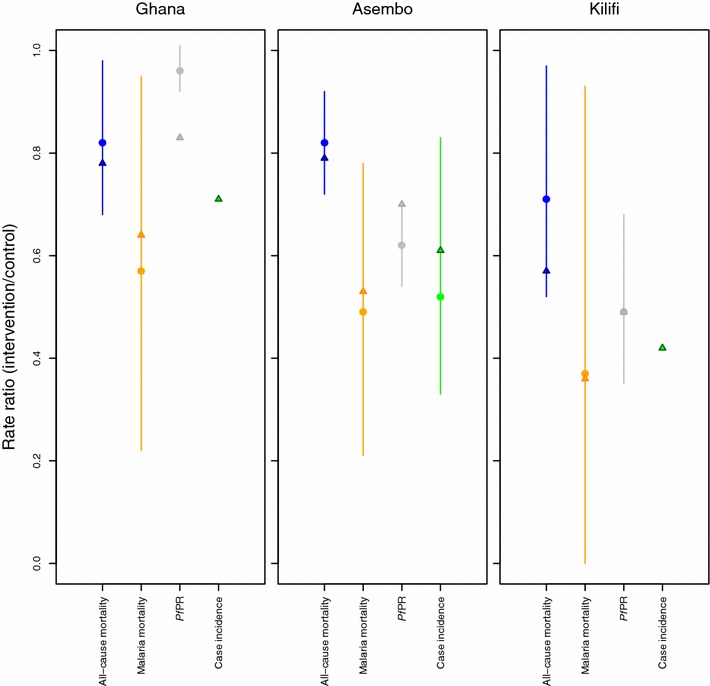
ITN impact on malaria outcomes: comparison between ITN trial observations and statistical model predictions. For calculation details and sources, see Additional file 3

Predicted proportional burden reductions in the ITN trials were generally comparable to those observed, with the largest proportional reductions for malaria mortality, followed by case incidence, and lesser reductions in *Pf*PR and all-cause post-neonatal under-5 mortality (Fig. 4; Additional file 3). In line with trial results, impacts were generally largest in Kilifi, followed by Asembo and Ghana, reflecting the ranking of the sites in baseline endemicity i.e. *Pf*PR (lowest in Kilifi, highest in Ghana). For nine of the ten health outcomes that could be evaluated across the three trials, the model-predicted relative risk was within the 95 % confidence interval (CI) of the observed data (Fig. 4). Simulations agreed with the trials in that *Pf*PR in Ghana had the smallest proportional reduction of all outcomes across the three trials, but the model-predicted reduction of 17 % in this outcome was outside the 95 % CI of the observed reduction (a point estimate of only 4 %).

### Sensitivity analysis

In out-of-sample predictions, regression models for health outcomes that had smaller simulated values, and thus more simulated zero outcomes (e.g. mortality in 15–99 years), generally gave higher MSE, higher ratio of MSE to variance, and lower R^2^.

Alternative regression models with outcome variables that dropped instead of imputed zero values, or that applied log instead of logit transformation on simulated outcomes, or that dropped the OpenMalaria model variant and simulated EIR as predictor variables, generally had lower R^2^ and higher MSE-to-variance ratios than the selected best models (Table 2).

**Table 2:** Statistical performance of selected and alternative statistical impact prediction models

Statistical model	Metric	Health burden outcome, and time period from intervention start			
		Case incidence 0–4 years, years 1–3	Case incidence 0–4 years, years 4–6	Malaria mortality 0–4 years, years 8–10	Malaria mortality 15+ years, years 8–10
Coefficient of variation^,^ i.e. ratio of standard deviation of the simulated distribution to the mean (does not depend on the statistical model)		125 %	129 %	138 %	279 %
Variance in simulated outcomes (does not depend on statistical model)		1.1	1.2	1.04e−4	3.1e−6
Selected (best) model: simulated 0 values imputed and remaining results re-scaled in the range 0–0.99	MSE, from out-of-sample prediction^a^	7.5 %	17.0 %	43.4 %	73.3 %
	Adjusted R^2^	96.5 %	92.7 %	90.3 %	74.1 %
	MSE as % of simulated variance	12.6 %	17.9 %	52.0 %	73.9 %
Simulated 0 values dropped and remaining results re-scaled in the range 0–0.99	Adjusted R^2^	96.6 %	92.2 %	86.8 %	70.1 %
	MSE as % of simulated variance	19.3 %	28.4 %	72.2 %	75.0 %
Dropping EIR and model variant (the two variables with no country data)	Adjusted R^2^	88.4 %	80.4 %	72.7 %	42.6 %
	MSE as % of simulated variance	75.1 %	406 %	142 %	89.8 %
Log-transformation instead of logit-transformation of health outcomes	Adjusted R^2^	96.0 %	93.0 %	90.6 %	77.1 %
	MSE as % of simulated variance	37.9 %	324 %	405 %	75.3 %

^a^Average of 25 simulations in which sub-samples of 100,000 simulations were randomly drawn to train and select the statistical model, and each time the remaining 65,888 simulations were used to assess its MSE

Nevertheless, across all alternative statistical models explored, the ranking of proportional burden reductions between interventions (for a given population coverage), between age groups, between health outcomes, between hypothetical Admin1 areas, and over time horizons was unchanged compared to the selected best model (Additional file 4). Excluding data with zero values made little difference to predicted risk ratios, with risk ratios varying between −6 and +2 % (median <0.0000 %) relative to the selected best model. Dropping EIR and Model Variant as predictor variables caused larger differences, with risk ratios varying −26 to +17 % (median −5 %) from the selected best model. The method of transformation had the largest influence, with risk ratios varying −51 to +17 % (median −6 %) from the selected best model.

## Discussion

### Methodology

This analysis shows that micro-simulations of complex patterns of health impacts following scale-up of malaria control in endemic African settings can be emulated by fitting fairly simple regression models to the simulated outputs. The emulations can use data on malaria endemicity and baseline intervention coverage to project the impact of alternative scale-up strategies for specific locations.

The high explanatory power of regressions attests to good internal validity against OpenMalaria simulations (Table 2; Additional file 2). The somewhat lower R^2^s for mortality outcomes may be explained by random noise in simulation results for mortality, since deaths are much rarer than positive infection status or cases. The imputation (or dropping) of zero simulation outcomes, the (logit) transformations applied to all outcomes before regression modelling, the re-scaling of health outcomes which did not naturally fall in the range 0–1 in order to allow logit transformation, and the implementation of predictor variables simulated in discrete steps as continuous in the regression (needed for predictions for a range of provinces) may have led to sub-optimally specified models. However, external and internal validities suggested that the potential bias introduced by this is minimal.

### Added value for programme planning projection tools

The impact functions thus developed for the Spectrum programme planning tool considerably improve on earlier malaria planning tools (notably the LiST child survival model), by: (i) predicting morbidity reductions, which accrue faster and are in the long term proportionally larger than mortality reductions; (ii) simulating different age groups, with proportional burden reductions in adults not much less than in young children; (iii) capturing variations in impacts over time, including partial rebounds. These rebounds result from the achieved endemicity reductions and consequent declines in acquired immunity, and become apparent from around 7 years after scale-up, in particular for mortality for people older than 5 years, as previously described in dynamic simulation studies of ITNs and SMC scale-up [23, 37, 38].

The incorporation of dependence of health impacts on baseline endemicity is another improvement. Modeled burden reductions are proportionally larger in settings with lower baseline malaria infection prevalence rates, and less seasonality in malaria transmission. This is consistent with observations from ITN trials [33] (Fig. 4) and with models of the dynamics induced by various malaria interventions [27, 39–41]. The absolute health gains—in terms of cases and deaths averted for a given coverage increase—are generally larger for higher-endemic settings, due to the larger baseline burden compared to lower-endemic settings. Existing programme planning tools, in contrast, have typically assumed fixed burden reductions at any time after intervention scale-up, in all countries and areas of Africa irrespective of endemicity.

Furthermore, the regression models capture non-linearity in the incremental health impact from progressive coverage increases, with some degree of saturation (diminishing returns) at high coverage levels. They also capture the synergy apparent in dynamic modelling studies [17] of higher-endemic settings between impacts of CM and ITNs.

Consistent with dynamic model-based assessments [30], impacts for a given population effective coverage level are larger for CM than for ITNs and IRS. However, it is often easier to achieve high-level coverage for vector control interventions (often delivered through vertical programmes, as campaigns) than for effective CM (through complex multi-layer health systems), so this ranking does not imply that CM is necessarily a better investment than vector control. The Spectrum-Malaria programme planning tool, by linking the current statistical effectiveness predictions with its costing module OneHealth Tool, will enable evaluation of both impacts and costs of malaria interventions and their trade-offs in short- and longer-term.

The models did not consider age differences in ITN and CM coverage, but these are likely to have only secondary effects since the burden reductions are partly driven by transmission effects which depend—especially in the longer term—mainly on average population-wide coverage and not just the coverage in people directly accessing the intervention.

There remains a need to refine these impact functions to incorporate drug and insecticide resistance, and extend them to impacts on *Plasmodium* species other than *P. falciparum*, such as *P. vivax* malaria (for countries with high prevalence of this species), which has very different dynamics from *P. falciparum* [42].

### Consistency with effectiveness data

These predictions of vector control impacts were generally consistent with best available data, as also used by WHO, the Roll Back Malaria partnership and international malaria donors [4, 34–36]. In particular, the predicted proportional burden reductions in young children following ITN scale-up were generally in line with those observed in cluster-randomized trials and other field studies and evaluations.

Also, the statistical predictions were consistent with recent ecological estimates of average ITN field impacts across sub-Saharan Africa based on synthesis of climatic, entomological, epidemiological and programmatic data across Africa, including larger proportional burden reductions at lower baseline *Pf*PR [2]. For malaria-related mortality in under five-year old children, the predicted 36–64 % reduction within 2 years for settings resembling the ITN trials in Kenya and Ghana, is similar to the estimate used in the LiST model of child survival of a fixed 55 at 100 % household ITN ownership (irrespective of endemicity or seasonality) [10, 11]. Predicted longer-term impacts were somewhat higher than the LiST time-fixed 55 % reduction, which reflects additional long-term transmission dynamic effects.

The external validation against trial data is complicated by imprecision and measurement challenges in ITN trial data: (i) The observed *case incidences* vary and are potentially biased across the trials by intensity of active case surveillance and treatment access, and the parasite density threshold used in case definition. (ii) The *infection prevalence* reductions depend strongly on heterogeneity in transmission [43]. If transmission is concentrated in a small subset of highly exposed individuals, then interventions will have little effect on prevalence, while if exposure is rather homogeneous, prevalence may be considerably reduced by the same intervention package. This can lead to deviations from the expected impacts in specific locations. (iii) For *mortality,* because of limitations in attributing child deaths (mostly in rural homes without medical confirmation) to malaria through verbal autopsy based on mostly a-specific symptoms, ITN trials focused on observed reductions in all-cause under-5 mortality. OpenMalaria simulated direct and indirect malaria-related deaths, but not other-cause deaths, and extrapolations from OpenMalaria predicted direct and indirect mortality reductions to either all-cause under-5 or reductions in malaria-attributable mortality involved some uncertain assumptions (Additional file 3).

The ITN and IRS type and effectiveness modelled were judged the most relevant generic representation of ITN and IRS as recommended for African programmes [44, 45]. The relative efficacies of ITNs and IRS reflect measurements from experimental hut studies using the insecticide Actellic CS recommended by the WHO’s Pesticide Evaluation Scheme for African programmes (for settings with mosquitoes fully susceptible to the insecticides used [18, 46]). These data drive the typically larger proportional burden reductions for ITNs than for IRS, which align with the recommendation of the WHO 2015 Global Technical Strategy to prioritize ITNs for vector control. However, absolute and relative effectiveness of ITN and IRS will vary among settings depending on local insecticide product choice (with the duration of protection sometimes less than the assumed 12 months), and site-specific insecticide resistance and ITN decay and usage and adherence patterns [47]. Current simulations and statistical functions did not capture these features, although they can be simulated in OpenMalaria through reduced effects on mosquito survival, and extent of personal protection [18]. There is also variation across Africa in the tendency of vector species to bite humans and to bite indoors. Highest ITN and IRS effectiveness and impact are expected in settings where highly anthropophilic (human biting) and indoor biting mosquitos (e.g. *Anopheles gambiae*, more so than *Anopheles arabiensis*) are responsible for most of the transmission.

For SMC, based on seven relevant trials in highly seasonal settings, the WHO has estimated a 75 % reduction in malaria case incidence, and considerable child mortality reduction within the year of implementation, but with unknown longer-term impacts [21]. The external validity of model results for SMC, therefore, remains to be assessed following future programme evaluations upon large-scale, long-term implementation.

The authors are not aware of any field studies of the effect of prompt and effective treatment of uncomplicated disease on incidence of severe malaria and mortality. Correspondingly, the models of the impact of scale-up of CM on burden are highly uncertain in OpenMalaria. Projections of impacts of scale-up of CM at country level face further uncertainty in estimates of effective coverage of treatment, which are typically available only from caregiver’s recall of treatment of febrile children under 5 years of age without stratification between malarial and non-malarial fevers, and without clear distinction of ineffective and effective treatment regimens [48, 49].

## Conclusions

In conclusion, predictions of health improvements following scale-up of malaria control interventions in varying *P. falciparum*-endemic areas in Africa, from computationally expensive transmission dynamic models, can to a large extent be emulated by regression models. The regression models developed in this study can be used to improve the simplistic effectiveness assumptions currently used in malaria programme planning and evaluation tools, despite substantial remaining uncertainties in the dynamic models underpinning the predictions.

## Additional files

10.1186/s12936-016-1461-9 Regression models of OpenMalaria-simulated malaria intervention impacts: predictor variable coefficients, with p values, and adjusted R^2^s.
10.1186/s12936-016-1461-9 Coefficients and p values of OpenMalaria-based statistical impact functions.
10.1186/s12936-016-1461-9 Simulation input assumptions and calculations on statistically predicted burden reductions for ITN trials.
10.1186/s12936-016-1461-9 Predicted relative burden reductions from alternative statistical models, as part of the sensitivity analysis.

## Abbreviations

AIC: Akaike’s information criterion
CM: effective case management
CV: coefficient of variation
EIR: entomological inoculation rate
GLM: generalized linear model(s)
IRS: indoor residual spraying
ITN: insecticide-treated mosquito net
LiST: lives saved tool (=child survival/mortality impact model)
MAP: malaria atlas project
MSE: mean squared error
*Pf*PR: prevalence of *Plasmodium falciparum* parasite infection
SMC: seasonal malaria chemoprophylaxis
WHO: World Health Organization
