# Supplementary material for: Malaria intervention scale-up in Africa: effectiveness predictions for health programme planning tools, based on dynamic transmission modelling
Source: Malar J. 2016 Aug 18;15:417. doi: 10.1186/s12936-016-1461-9 (PMC4991118; doi:10.1186/s12936-016-1461-9)
Supplement: Supplementary file 1 — 10.1186/s12936-016-1461-9 Regression models of OpenMalaria-simulated malaria intervention impacts: predictor variable coefficients, with p values, and adjusted R2s. [file 12936_2016_1461_MOESM1_ESM.docx]

Korenromp-EL et al., *Malaria intervention scale-up in Africa: effectiveness predictions for health program planning tools, based on dynamic transmission modelling, version 01 May 2016*

# Additional File 1: Regression models of OpenMalaria-simulated malaria intervention impacts: predictor variable coefficients, with p-values, and adjusted R^2^s

Health burden outcomes were modelled in the logit scale. Outcomes such as case incidence or mortality rates that can take values greater than one were first re-scaled, by dividing them by the maximum value across all 165,888 simulations, multiplied by 1.2. This ensured that natural constraints to these outcomes were preserved, e.g. rates remained positive and prevalence rates stayed in the rage 0 to 1. To be more specific: to model a given outcome z at the time horizon t, let $y=z/c$, where $c=\max\left( Y \right)/0.99$, and Y is the vector of observed outcome.

We assumed that the transformed burden outcomes follow a linear model given by (Eq1.1), where *a* indicates the age group, *v* denotes the independent variables (Table below).$\beta_{a0t}$ is the intercept, $\beta_{avt}$,$\gamma_{avt}$, and $\theta_{av1:2t}$ are the respective coefficients of the first order, second order and interaction (between $v1$ and $v2$) terms, *i* is the simulation number, $V_{vi}$ is the value taken by the variable $V$ for *i*, $\varepsilon_{ati}$ is the error term, $f\left( y \right)=log(\frac{y}{1-y})$.

$f\left( y_{a,t,i} \right)=\beta_{a0t}+\sum_{v} \beta_{avt}V_{vi}+\sum_{v\neq v_{m}} \gamma_{avt}V_{vi}^{2}+\sum_{v1\neq v2} \theta_{av1:2t}V_{v1i}:V_{v2i}+\varepsilon_{ati} Eq1.1$

where $v_{m}$ represent the OpenMalaria model variant (the only categorical predictor variable) and for which the squared term is not defined.

Parameters were estimated by minimizing the least squared error and model selection was performed using Akaike’s Information Criterion (AIC) for the linear model described by Eq1.1.

The impact functions were obtained after the fitting procedure. OpenMalaria model variant and 2000–2002 average simulated EIR were, however, included in regression models for all burden outcomes. Because these variables are not known at country and province levels for impact predictions in practice, we developed another model to predict from pre-2016 simulation parameters and results (see Annex 1 below) the simulated EIR averaged over 2000–2002. We then applied that predicted EIR in the impact prediction, predicting y for each of the six OpenMalaria model variants using (Eq.1.2)

$$\hat{y}_{a,t,i}\left| V_{v_{m}} \right.=f^{-1}\left( \hat{\beta}_{a0t}+\sum_{v} \hat{\beta}_{avt}V_{vi}+\sum_{v\neq v_{m}} \hat{\gamma}_{avt}V_{vi}^{2}+\sum_{v1\neq v2} \hat{\theta}_{av1:2t}V_{v1i}:V_{v2i} \right) Eq1.2$$

where $f^{-1}(y)=\exp\left( y \right)/(1+\exp\left( y \right))$ ; hats were added to distinguish estimated coefficients though they have the same meaning as in Eq1.1. We set coefficients to zero when the corresponding term was not selected based on AIC. Finally, we obtained the predicted outcome by taking the average over the model variants, assuming that model variants are equally weighted:

$$\hat{z}_{a,t,i}=cE\hat{y}_{a,t,i}\left| V_{v_{m}} Eq1.3 \right.$$

See further: *AddFile2_Coefficients+P-values OpenMalaria-based statistical Impact models_Korenromp et al_29April2016.XLS .*

**Annex 1, within Additional File 1:
Prediction of the pre-intervention EIR (2000–2002)**

The aim of our statistical modelling was to describe health burden reduction for settings differing in baseline malaria endemicity and control situation, and apply that to predict impacts of malaria control for African provinces and countries based on available health outcome indicator data. However, the OpenMalaria simulations underlying the statistical models varied the annual EIR as a key parameter, for which no routine data or standardized estimates are available at country and province levels, and the OpenMalaria calibration variant (called ‘model variant’) which reflects uncertainty in malaria natural history and human acquired immunity, but is not a feature specific to countries or provinces. However, excluding these two predictor variables in the statistical models would amplify the noise and, because our transformations do not preserve the mean, potentially bias predicted burden levels and reductions. Therefore, these two predictor variables were included in the impact models, operationalized for EIR, analogous to the *Pf*PR predictor variable, as the average simulated EIR over 2000–2002 (which differs from the design EIR shown in Table 1, as simulated EIR varies with the baseline coverage of IRS). To impute the 2000–2002 EIR predictor value for predictions for provinces that lack EIR data, we fitted a regression similar to the one presented in Eq1, using 2000–2002 EIR as dependent variable:

$$log10(EIR)=\beta_{a0}+\sum_{v} \beta_{av}V_{vi}+\sum_{v} \gamma_{av}V_{vi}^{2}+\sum_{v1\neq v2} \theta_{av1:2}V_{v1i}:V_{v2i}+\varepsilon_{ati} Eq1.4$$

Here, the independent variables were (i.e. $v$ belongs to) the set made of pre-intervention variables, i.e. $PfPR\_2000\_2002$, $\left( PfPR\_2000\_2002 \right)^{1/3.5}, \left( PfPR\_2000\_2002 \right)^{-1/4.5}$, $\left( PfPR\_2000\_2002 \right)^{2/5}$, seasonality CV covInitIRS, and covInitCM (see Table SI-1). All these variable were statistically significant (P<0.05) and the resulting coefficient of determination (R^2^) was about 98% (see:

AddFile1_Coefficients+P-values OpenMalaria-based statistical Impact models_08April2016).
