# Supplementary material for: Malaria intervention scale-up in Africa: effectiveness predictions for health programme planning tools, based on dynamic transmission modelling
Source: Malar J. 2016 Aug 18;15:417. doi: 10.1186/s12936-016-1461-9 (PMC4991118; doi:10.1186/s12936-016-1461-9)
Supplement: Supplementary file 3 — 10.1186/s12936-016-1461-9 Simulation input assumptions and calculations on statistically predicted burden reductions for ITN trials. [file 12936_2016_1461_MOESM3_ESM.docx]

Korenromp-EL et al., *Malaria intervention scale-up in Africa: effectiveness predictions for health program planning tools, based on dynamic transmission modelling, version 01 May 2016*

# Additional File 3. Simulation input assumptions and calculations on statistically predicted burden reductions for ITN trials

Table AF3.A shows values of predictor variables assumed in statistical predictions of ITN impact, for the three cluster-randomized trials of ITN impacts on child health, evaluated as a test to the external validity of regression models.

**Table AF3.A Predictor values assumed in statistical predictions of ITN impact for cluster-randomized trials**

| Trial location | *Pf*PR, Control arm | Seasonality CV | ITN coverage/ usage last night | | Coverage of effective treatment, uncomplicated cases | | | | |  |
| --- | --- | --- | --- | --- | --- | --- | --- | --- | --- | --- |
|  |  | |  | C arm | | I arm | Pre-trial (C and I arms) | C arm, trial | I arm, trial | |
|  | Predictions | Trial data |  |  | |  |  |  |  | |
| Upper-East province, Ghana | 69%*  (0-59 months) | 69%  (0-59 months) | 1.53 | 0% | | 60% | 10% | 10% | 10% | |
| Asembo Bay, Western Kenya | 48%*  (0-59 months) | 63%  (0–35 months, 3^rd^/ last survey) | 0.50 | 5% | | 57% | 10% | 30%** | 30%** | |
| Kilifi / Coast, Kenya | 35%  (2–9 years) | 35%  (1–9 years) | 0.48 | 0% | | 60% | 10% | 30%** | 30%** | |
| Source | * | [[1](#_ENREF_1)] | [[2](#_ENREF_2), [3](#_ENREF_3)] | Asembo: [[4](#_ENREF_4), [5](#_ENREF_5)]  Ghana: [[6](#_ENREF_6)]  Kilifi: [[7](#_ENREF_7)] | | | | | | |

Abbreviations: CV = coefficient of variation, C = control, I = intervention; ITN = insecticide treated net.

Notes to Table AF3.A:

*** Calculated from predictions for 2-9 year group, using age patterns and relationship as in OpenMalaria simulations:
$y= 0.282863x+ 0.72199x^{2}$,
where $y$ is *Pf*PR in the age group 0 to 4 years, and $x$ is *Pf*PR in the age group 2 to 9 years.

In the two Kenya trials, coverage of effective case management was higher than the regional typical coverage at the time, since trials undertook active disease surveillance and treated any detected cases, in both control and intervention arms. These trial-specific high treatment coverages were simulated (instead of provincial averages for the pre-ITN period), to avoid over-estimating the added value of ITNs.

Our external validation evaluated the regression models ‘fit’ against trial-observed reductions for both for all-cause post-neonatal under-5 mortality and for malaria-attributable mortality (MAM). These two validation outcomes are based on the same trial-observed data for all-cause post-neonatal under-5 mortality (ACCM), but expressed differently. In trial data, the protective efficacy (PE) of ITNs in reducing malaria-attributable mortality (the complement of the rate ratio shown) reflects the observed ACCM rate reduction, expressed as percentage of control-arm malaria-attributed mortality, according to the definition used by WHO and the UN Child Health Epidemiology Reference Group (CHERG) in regional and global estimates of malaria mortality trends and lives saved by ITNs [[8](#_ENREF_8)]:

$${PE}_{MAM}=\frac{{PE}_{ACCM}}{\beta}$$

where $\beta$ is an estimate of the proportion of deaths from all-causes due to malaria among children in areas without access to malaria prevention interventions, which was estimated at 36% in Asembo [[4](#_ENREF_4), [5](#_ENREF_5)], 41% in Ghana [[6](#_ENREF_6)], and 46% in Kilifi [[7](#_ENREF_7)].

OpenMalaria outputs and their statistical model emulations do not provide estimates of ACCM, but instead, provide estimates of direct and indirect malaria mortality in ITN and control arms. In order to convert these outputs to estimates of the risk ratio of ITN intervention in terms of ACCM and MAM in the single-cause of death framework used by MERG, the equations below for the risk ratios for MAM (RR_MAM_; Equation 3.1) and ACCM (RR_ACCM_; Equation 3.2) were used:

${RR}_{MAM}=\frac{{DMR}_{i}}{{DMR}_{c}+{IMR}_{c}-{IMR}_{i}}\mathrm{Eq}3.1$

with:

DMR_i_ the direct malaria mortality rate in the intervention arm,

DMR_c_ the direct malaria mortality rate in the control arm,

IMR_i_ the indirect malaria mortality rate in the intervention arm,

IMR_c_ the indirect malaria mortality rate in the control arm.

${RR}_{ACCM}=\frac{{DMR}_{i}+{IMR}_{i}+{ACCM}_{c}\left( 1-\beta\left( 1+\frac{{IMR}_{c}}{{DMR}_{c}} \right) \right)}{{DMR}_{c}+{IMR}_{c}+{ACCM}_{c}\left( 1-\beta\left( 1+\frac{{IMR}_{c}}{{DMR}_{c}} \right) \right)()} \mathrm{Eq}3.2$

with:

ACCM_c_ the ACCM from the trial data control arm,

DMR_i_ the direct mortality in the intervention arm,

DMR_c_ the direct mortality in the control arm,

IMR_i_ the indirect mortality in the intervention arm,

IMR_c_ the indirect mortality in the control arm,

$\beta$ the estimated proportion of deaths from all-causes due to malaria among children in areas without access to malaria prevention interventions, as above.

In Equation 3.1, the denominator is the direct malaria mortality in the control arm plus the difference in indirect mortality between control and intervention arms, i.e. indirect malaria-related mortality that would be identified as due to malaria in the single cause of death framework because it was prevented by the intervention. This corresponds to the definition and calculation of RR_MAM_ used by the United National Child Health Epidemiology Reference Group (CHERG) used as input to the LiST model of child survival interventions [[8](#_ENREF_8)].

Equation 3.2 calculates the corresponding RR_ACCM_ in OpenMalaria-based regressions, as the sum of the model-predicted malaria direct mortality rate in the intervention arm, the model-predicted indirect malaria-related mortality rate in the intervention arm and the non-malaria (other-cause) mortality rate in the control arm in trial observations (which was assumed to be the same in the trial intervention arm, and in predictions), divided by the sum of model-predicted malaria direct mortality in the control arm, model-predicted indirect malaria-related mortality in the control arm and non-malaria (other-cause) mortality in the control arm in trial observations.

Using these equations, Table AF3.B shows predictions against the data with their 95% confidence bounds, illustrating that for 9 of 10 health outcomes evaluated across the three trials, the predicted relative risk was within the 95% confidence bound of the trial observation. The one consistent difference is *Pf*PR in Ghana.

**Table AF3.B Observed and regression-predicted relative risks (intervention / control arm) of health outcomes in 3 cluster-randomized ITN trials**

| Trial | Health outcome | Observed | |  | Predicted | Comments |  |
| --- | --- | --- | --- | --- | --- | --- | --- |
|  |  |  | Low-bound | Upper-bound |  |  | |
| Navrongo, GHANA | All-cause post-neonatal under-5 mortality | **0.82** | 0.68 | 0.98 | **0.78** | Data from Cochrane review [[1](#_ENREF_1)] | |
|  | Malaria-attributable mortality | **0.57** | 0.22 | 0.95 | **0.64** | Calculated as described in text of Additional File 3 | |
|  | Malaria case incidence | **NA** |  |  | **0.71** |  | |
|  | PfPR | **0.96** | 0.92 | 1.01 | **0.83** | Data from Cochrane review [[1](#_ENREF_1)] | |
|  |  |  |  |  |  |  | |
| Asembo, KENYA | All-cause post-neonatal under-5 mortality | **0.82** | 0.72 | 0.92 | **0.79** | Data based on hazard ratio of 0.72, adjusted for community contamination* | |
|  | Malaria-attributable mortality | **0.49** | 0.21 | 0.78 | **0.53** | Calculated as described in text of Additional File 3 | |
|  | Malaria case incidence | **0.52** | 0.33 | 0.83 | **0.61** | Data based on hazard ratio of 0.52, adjusted for community contamination* | |
|  | PfPR | **0.62** | 0.54 | 0.70 | **0.70** | Data based on hazard ratio of 0.59, adjusted for community contamination* | |
|  |  |  |  |  |  |  | |
| Kilifi, KENYA | All-cause post-neonatal under-5 mortality | **0.71** | 0.52 | 0.97 | **0.57** | Data from Cochrane review [[1](#_ENREF_1)] | |
|  | Malaria-attributable mortality | **0.37** | 0 | 0.93 | **0.36** | Calculated as described in text of Additional File 3 | |
|  | Malaria case incidence | **NA** |  |  | **0.42** |  | |
|  | PfPR | **0.49** | 0.35 | 0.68 | **0.49** | Data from Cochrane review [[1](#_ENREF_1)] | |

* Community contamination-adjusted Hazard Ratios (HR) from [[5](#_ENREF_5)]; converted into relative risks (RR) using the formula: $RR=\frac{HR}{1+Prevalence\left( HR-1 \right)}$ with prevalence denoting the *Pf*PR or mortality or case incidence in the comparison arm.

#### References for Additional File 3:

1. Lengeler C: **Insecticide-treated bed nets and curtains for preventing malaria**. In: *Cochrane Database Syst Rev.* Edited by Group EGCID; 2004: CD000363.

2. Sinka ME, Bangs MJ, Manguin S, Coetzee M, Mbogo CM, Hemingway J, Patil AP, Temperley WH, Gething PW, Kabaria CW *et al*: **The dominant Anopheles vectors of human malaria in Africa, Europe and the Middle East: occurrence data, distribution maps and bionomic precis**. *Parasit Vectors* 2010, **3**:117.

3. Sinka ME, Bangs MJ, Manguin S, Rubio-Palis Y, Chareonviriyaphap T, Coetzee M, Mbogo CM, Hemingway J, Patil AP, Temperley WH *et al*: **A global map of dominant malaria vectors**. *Parasit Vectors* 2012, **5**:69.

4. Phillips-Howard PA, Nahlen BL, Kolczak MS, Hightower AW, Ter Kuile FO, Alaii JA, Gimnig JE, Arudo J, Vulule J, Odhacha A *et al*: **Efficacy of permethrin-treated bednets in the prevention of mortality in young children in an area of high perennial malaria transmission in western Kenya**. *Am J Trop Med Hyg* 2003, **68**(Suppl.04):23-39.

5. Hawley WA, Phillips-Howard PA, ter Kuile F, Terlouw DJ, Vulule JM, Ombok M, Nahlen BL, Gimnig JE, Kariuki SK, Kolczak MS *et al*: **Community-wide effects of permethrin-treated bednets on child mortality and malaria morbidity in Western Kenya**. *Am J Trop Med Hyg* 2003, **68**(Suppl.4):121-127.

6. Binka FN, Kubaje A, Adjuik M, Williams LA, Lengeler C, Maude GH, Armah GE, Kajihara B, Adiamah JH, Smith PG: **Impact of permethrin impregnated bednets on child mortality in Kassena-Nankana district, Ghana: a randomized controlled trial**. *Trop Med Int Health* 1996, **1**(2):147-154.

7. Nevill CG, Some ES, Mung'ala VO, Mutemi W, New L, Marsh K, Lengeler C, Snow RW: **Insecticide-treated bednets reduce mortality and severe morbidity from malaria among children on the Kenyan coast**. *Trop Med Int Health* 1996, **1**(2):139-146.

8. Eisele TP, Larsen D, Steketee R: **Protective efficacy of interventions for preventing malaria mortality in children in *Plasmodium falciparum* endemic areas / Modeling the Impact of Scaling up Interventions for Malaria**. *Int J Epid* 2010, **39**(Suppl.1):i88-i101.
